# Evaluation of HIV-1 rapid tests and identification of alternative testing algorithms for use in Uganda

**DOI:** 10.1186/s12879-018-3001-4

**Published:** 2018-02-27

**Authors:** Pontiano Kaleebu, Paul Kato Kitandwe, Tom Lutalo, Aminah Kigozi, Christine Watera, Mary Bridget Nanteza, Peter Hughes, Joshua Musinguzi, Alex Opio, Robert Downing, Edward Katongole Mbidde

**Affiliations:** 1 Uganda Virus Research Institute, Entebbe, Uganda; 2 MRC/UVRI Uganda Research Unit, Entebbe, Uganda; 3 London School of Hygiene and Tropical Medicine, London, UK; 4 UVRI-IAVI HIV Vaccine Program, Entebbe, Uganda; 5 Rakai Health Sciences Programme, Entebbe, Uganda; 6 AIDS Control Programme, Ministry of Health, Kampala, Uganda

**Keywords:** HIV-1, Rapid tests, Weak bands, Evaluation, Algorithms, Uganda

## Abstract

**Introduction:**

The World Health Organization recommends that countries conduct two phase evaluations of HIV rapid tests (RTs) in order to come up with the best algorithms. In this report, we present the first ever such evaluation in Uganda, involving both blood and oral based RTs. The role of weak positive (WP) bands on the accuracy of the individual RT and on the algorithms was also investigated.

**Methods:**

In total 11 blood based and 3 oral transudate kits were evaluated. All together 2746 participants from seven sites, covering the four different regions of Uganda participated. Two enzyme immunoassays (EIAs) run in parallel were used as the gold standard. The performance and cost of the different algorithms was calculated, with a pre-determined price cut-off of either cheaper or within 20% price of the current algorithm of Determine + Statpak + Unigold. In the second phase, the three best algorithms selected in phase I were used at the point of care for purposes of quality control using finger stick whole blood.

**Results:**

We identified three algorithms; Determine + SD Bioline + Statpak; Determine + Statpak + SD Bioline, both with the same sensitivity and specificity of 99.2% and 99.1% respectively and Determine + Statpak + Insti, with sensitivity and specificity of 99.1% and 99% respectively as having performed better and met the cost requirements. There were 15 other algorithms that performed better than the current one but rated more than the 20% price. None of the 3 oral mucosal transudate kits were suitable for inclusion in an algorithm because of their low sensitivities. Band intensity affected the performance of individual RTs but not the final algorithms.

**Conclusion:**

We have come up with three algorithms we recommend for public or Government procurement based on accuracy and cost. In case one algorithm is preferred, we recommend to replace Unigold, the current tie breaker with SD Bioline. We further recommend that all the 18 algorithms that have shown better performance than the current one are made available to the private sector where cost may not be a limiting factor.

## Background

Rapid tests (RTs) for HIV antibody in blood and saliva are being promoted for HIV diagnosis in resource limited settings in order to improve the quality of service delivery, acceptability and uptake of HIV testing and counselling (HTC) [1, 2]. The advantage of RTs compared with enzyme immunoassays (EIA) is the simplicity in use and the quick turn-around time of results leading to improvements in service delivery including prevention, care and treatment. Unlike EIAs, RTs do not require much use of instrumentation and with good training can be used by non-laboratory staff in remote centres. In the past few years, there has also been an increasing interest in the use of HIV self-testing (HIVST) to help increase testing coverage and a number of countries already have HIVST policies [3]. HIVST is where an individual, who wants to know his or her HIV status collects his/her specimen, tests it and interprets the results, often in private. Most HIVST is by the use of oral tests that use saliva and currently one HIVST kit, OraQuick In-Home HIV Test is approved by the USA Food and Drug Administration (FDA) [4]. Furthermore, there are now 4th generation RTs that allow detection of infection in the earlier phase of infection due to the ability to detect HIV antigen which appears earlier than antibodies after infection. The challenge is that some of these 4th generation RTs are still lagging behind, largely due to their inadequate performance so far especially in detecting acute infections [5, 6], and only the Determine HIV 1/2 Ag/Ab Combo is currently approved by the FDA.

The Joint United Nations Programme on HIV/AIDS (UNAIDS) 90–90-90 targets aim at 90% of people knowing their HIV status, 90% of those positive receiving ARVs and 90% of those receiving ARVs suppressing viral load to below detection by 2020 [7]. The attainment of this will require unprecedented roll out of HTC. Nations will need to ensure HTC expansion to meet these targets but also to provide quality HIV testing as a priority.

In Africa, various RT kits are currently on market and different algorithms are being used, but evaluation of these tests especially in field settings of the general populations has been limited [8]. In addition, most HIV test kits have been evaluated as individual kits and not testing algorithms. The global practice is that each country must select and standardize their testing algorithms based on local situations e.g. costs, storage issues, human resource, infrastructure, service demand etc. [9].

In Uganda, HIV rapid testing began in 1990 using Capillus HIV-1/HIV-2 (Cambridge Diagnostics) and since then various kits have been used [10, 11], it was in 2006 that a national testing algorithm of Determine HIV 1/2, HIV-1/2 Statpak and Uni-Gold HIV was introduced but without extensive evaluation. Other algorithms have been proposed [12] but their evaluations were based on limited kits and without following the recommended World Health Organization (WHO) and US Center for Disease Control and Prevention (CDC) guidelines [8, 13]. One other challenge with the current national algorithm is related to the interpretation of results that show weak positive (WP) bands which have led to low specificity and positive predictive value (PPV) [14]. With more kits on market, there is therefore a need to evaluate them, come up with better algorithms for the country and make recommendations on the observed WP bands. The objectives of this study therefore were to determine the appropriate HIV RT and recommend 2–3 algorithms to be used in Uganda.

Specific objectives were 1) To assess the performance of eleven blood and three oral fluid based rapid tests and come up with best algorithms. 2) To determine the proportion of HIV positive RTs with WP bands but which eventually turn out negative on confirmation using EIA. 3) To ascertain the inter-reader agreement in HIV diagnosis using RTs. 4) To assess the correlation of finger stick blood HIV results and those obtained by use of plasma.

## Method

The study followed the CDC/WHO guidelines for RT evaluations [13]. It was a two phase study where phase I was a laboratory-based evaluation to determine test performance and to identify 3 algorithms that were later evaluated in phase II at the point of care (POC). Phase II used a finger prick by laboratory and non-laboratory staff and quality control (QC) at the National Reference Laboratory (NRL) performed on 10% of the negative and 100% of positive samples using the algorithms used at the POC. For oral based kits, both laboratory and field testing took place in phase I.

A total of eleven blood based kits were evaluated namely: Determine HIV1/2, HIVSav 1&2 rapid serotest, Acon HIV 1/2/0 Triline, Uni-gold HIV, SD Bioline HIV 1/2 3.0, First Response HIV 1–2. O, Carestart HIV 1.2.O, Doublecheck Gold HIV 1&2, HIV 1/2 StatPak, Medinostics HIV 1/2 Gold rapid screen and Insti HIV antibody. The three oral transudate kits evaluated were OraQuick HIV-1/2, Calypte Aware HIV-1/2 OMT and Chembio DPP HIV1/2.

These were selected from the United States Agency for International Development (USAID) list of approved HIV kits. These kits had undergone evaluation and approval by either FDA or another stringent regulatory approval for Canada, European Union Japan or USAID. From this list, kits that did not meet the study’s selection criteria of storage (2–30^0^ C), specimen type to use serum, plasma and whole blood, ease of use (that included number of steps, ease of reading the test, nature of specimen collection and whether no special equipment was required), and shelf life (at least 12 months) were excluded. Other reasons for kits exclusion were limited information and not for our Ugandan market.

Before the study, the study team was trained on the standard operating procedures (SOPs) and on the protocol. Site visits were made in preparation for and during the study to monitor progress. Ten milliters (mls) of venous blood was collected in ethylenediaminetetraacetic acid (EDTA) (5 ml), the anticoagulant preferred by most test kit manufacturers and serum separating tubes (SST 5 ml). The blood based kits were evaluated using EDTA whole blood while oral kits were evaluated using oral transudate by swabbing of participants gums to obtain sample. Each kit was tested following the instructions in its insert. The serum from the SST tubes was used for confirmatory testing using 2 EIAs i.e. Vironostika HIV Uni-form II plus O and Murex HIV-1.2.O run in parallel as the gold standard. In-house cut off values used by CDC Uganda were used (UVRI NRL QC/quality assurance (QA) report 2000). These cut offs had been established after validation of these EIAs using local samples in order to increase their specificity without compromising sensitivity. The EIAs were done at Medical Research Council/UVRI Uganda at Entebbe and at Rakai Health Sciences Program (RHSP) in Rakai with technicians blinded to the RT results.

Testing of the blood based kits was done by laboratory technicians at the laboratories at each site while oral transudate kits were evaluated by nurse counsellors at the respective sites. In order to address objective number two, results were scored as weakly reactive or WP when the intensity of the test band was weaker than the control band but in the calculation of kit performance, all test bands were interpreted as positive regardless of intensity as required by the manufacturer. All results were read independently by two operators at three sites in order to assess inter-observer agreement.

The sensitivity (Se), specificity (Sp), negative predictive value (NPV), PPV false positive rate (FPR) and false negative rate (FNR) of each kit was calculated using the EIAs as gold standard. The performance criteria for the desirable algorithms were then established. A minimum sensitivity of 99.0% was set for the screening kits and a minimum specificity of 98.0% was set for the confirmatory and tie breaker kits [2]. Combinations of all kits that met the above criteria were then made and the performance (Se, Sp, PPV, NPV, FPR, FNR) and cost of the different algorithms calculated.

The cost of the kits was determined using the HIV Test kits listed in the USAID Source and Origin Waiver [15]. We also compared this with the WHO procurement prices for 2013. The cost of each algorithm was estimated using the formula described in Appendix A using the average national prevalence of 7.3% [16]). The technical team of the ministry proposed a new algorithm had to be cheaper or within 20% price of the current algorithm. The price of the algorithm being used at the time was about 1.50 US$ per test. Results were double entered and consistency checks done at UVRI.

### Study sites and participant selection

**Fig. 1:**
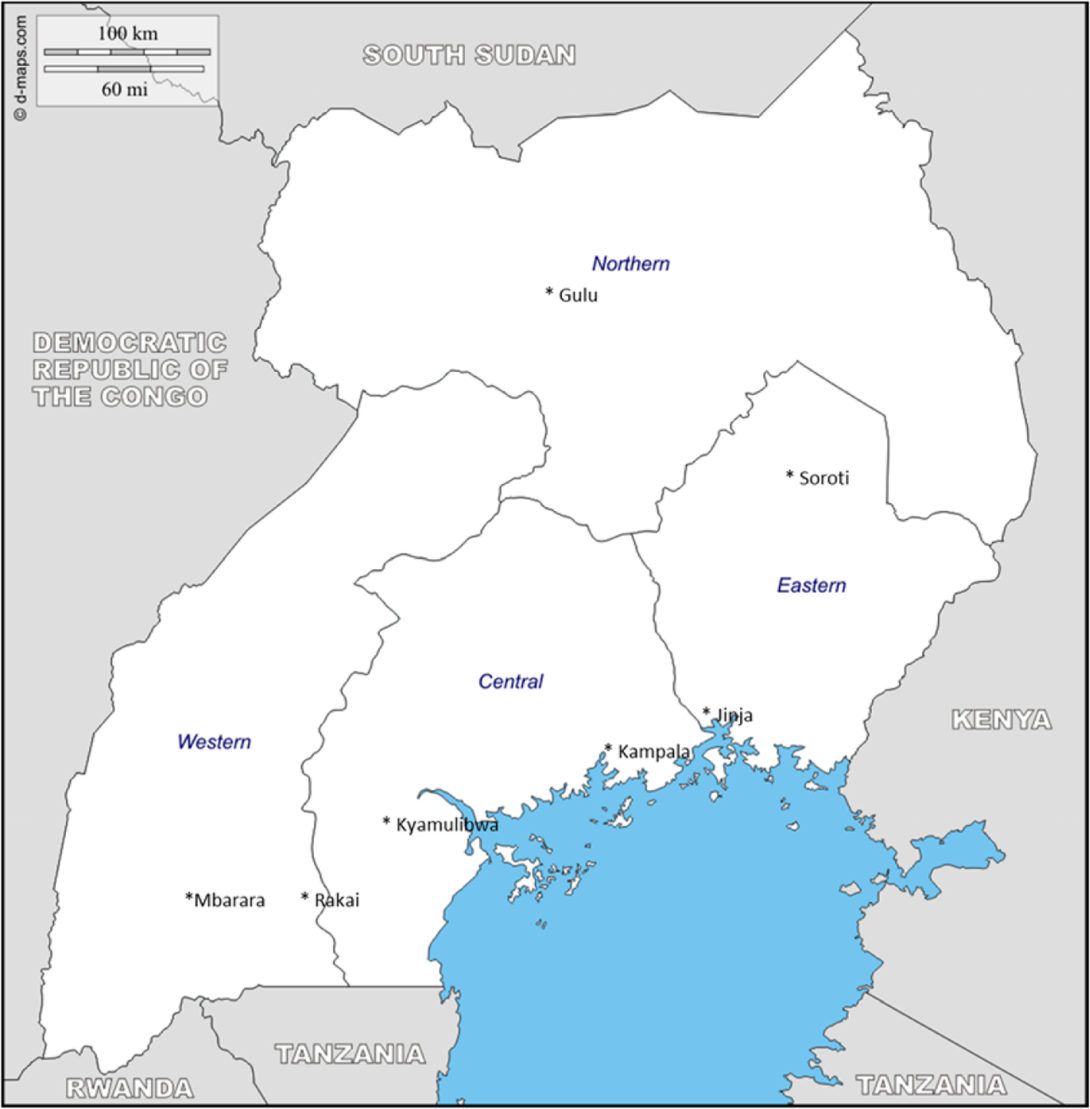
Map of Uganda showing the four regions and site locations where the phase I testing was performed. Source: http://d-maps.com/carte.php?num_car=4025&lang=en but with the study sites added

Seven sites, covering the four different regions of Uganda, participated in the phase I evaluation namely; − AIDS Information Centre (AIC) Kampala and MRC/UVRI Kyamulibwa (Central), Rakai Health Sciences Program (RHSP) and Mbarara Regional Referral Hospital (Western), St. Mary’s Lacor Hospital, Gulu (Northern), The AIDS Support Organization (TASO) Jinja and TASO Soroti (Eastern), see site locations in Fig. 1. Participants already known to be HIV positive were recruited after collection of the 200 HIV negative samples at each site. Four sites AIC, TASO Entebbe, TASO Masaka and Lacor Hospital participated in phase II.

### Statistical methods

#### Sample size

The study followed the CDC/WHO guidelines for HIV test evaluation [13] which recommend that a test evaluation exercise should have a minimum of approximately 200 HIV-positive and 200 HIV negative specimens to provide 95% confidence intervals of less than ±2% for both the estimated sensitivity and specificity. This number was considered at each of the 7 study sites (Rakai, Kyamulibwa, AIC, Tororo, Jinja, Mbarara and Gulu) to give a targeted enrolment of 2800 individuals (1400 HIV negatives and 1400 HIV positives). Consideration of this sample size per region was decided to give an opportunity to investigate whether there are environmental or other local factors that could affect test performance, especially the reported weak positive bands.

#### Samples included and those excluded in the analysis

A total of 200 HIV positive and 200 HIV negative individuals from each of the sites involved were eligible. However, the analysis only considered those individuals whose samples were in quantities that would allow for all the tests under evaluation together with EIA tests to be performed. Those that did not have enough samples were excluded from the analysis. The analysis also excluded those that had discordant EIA tests.

#### Dates of sample collection for phase I

The samples were drawn from October 2010 to December 2012. More specifically, for Lacor, this was 4th October 2010 – 11th February 2011. MRC Kyamulibwa, 7th October 2010 - 5th October 2011; Mbarara: 14th October 2010 - 17th December 2010; RHSP: 12th October 2010 - 16th February 2011. AIC-Kampala: 23rd November 2010 - 15th April 2011; Jinja: 4th January 2011 - 13th December 2012 and Soroti: 13th September 2011 - 9th May 2012.

#### Dates of sample collection for phase II

TASO Masaka was 5th February 2015 to 11th March 2015; TASO Entebbe 25th February 2015 to 30th March 2015; Lacor Hospital 18th February 2015 to 20th April 2015 and AIC was 13th April 2015 to 29th June 2015.

#### To assess specificity, sensitivity, negative and positive predictive values

STATA 12 was used to calculate the sensitivity, specificity PPV and NPV. These values were calculated by first taking the WP results as positive and then removing the WP results from the calculation to determine the influence of WPs on false positive rates.

#### Receiver operating curves (ROC)

Though our major interest was Se, Sp, NPV and PPV, we used the STATA command roccomp to compare the ROC generated using the three best algorithms to the current national algorithm in use (Determine + Statpak + Unigold).

#### Developing an algorithm

Data were analyzed to determine the performance of individual tests. Kits that passed the above sensitivity criteria were considered as screening tests while kits that passed the specificity criteria were considered as confirmatory tests or as a tie breaker when evaluating an algorithm. For the algorithm evaluation, the study considered a serial testing model whereby specimens were considered as true negative if they reacted negatively on the first test. For specimens reactive on the first test a confirmatory test would be considered. If specimens were concordantly positive by the two assays, they were considered as true-positives. For discordantly reactive sera, the third test kit or tie breaker results in the algorithm would be considered as definitive.

#### To determine the proportion of HIV positive RTs with WP bands but which eventually turn out negative on EIA confirmation

In order to assess whether the WP scores were equally distributed in the four geographical regions for each RT, cross tabulation of the blood RT results against the EIA tests were generated indicating the percentage of WPs that returned positive or negative EIA results.

#### To ascertain the inter-reader variability using RTs

In order to determine the inter-reader variability of the RTs, two persons independently interpreted each test result, and the reader variability, expressed as percentage of sera for which different readers interpret test results differently was calculated for each RT.

#### Quality assurance

This study was conducted under a rigorous QA program which included among others, training of personnel, development of SOPs, coordination of sample transport and storage, equipment maintenance and proficiency testing. Samples were given unique identifiers in all the laboratories.

##### Phase II

In the second phase, the three algorithms selected in phase I were used in the field, at the POC for purposes of QC. Finger stick whole blood testing was done at three sites where two different algorithms per site were used. Matching plasma from all HIV positive specimens and 10% of HIV negative specimens were retested at the NRL at UVRI using the same testing algorithms as at the POC.

Before initiation of phase II, there was a change in the manufacture of Determine HIV1/2 from Abbott to Alere Medical Co Ltd., Japan. Although the kit composition did not change, we compared the performance of both kits and showed these two kits were not different in their performance (*results not included)*.

## Results

**Fig. 2:**
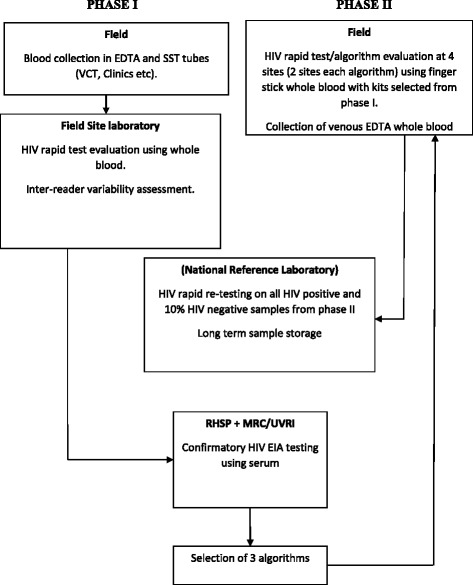
The logic framework for phase I, the laboratory-based evaluation and phase II at the point of care

Figure 2 shows the logic framework for phase I and II.

### Phase I

In phase I, a total of 2800 participants were enrolled, with 400 samples tested at each of the 7 centers. However, the kits were evaluated using samples from 2746 participants i.e. 392 from AIC; Mbarara 387; Kyamulibwa 392; Rakai 399; Lacor 384; Jinja 395 and Soroti 397 participants. Those missing had either EIA test results that were discordant or samples collected were insufficient for the Gold standard test.

First Response and Carestart had more WP results; they scored 36.7% and 36% respectively. These were followed by Acon Triline (27.5%) and HIVSav (25.7%). StatPak (3.0%) followed by Determine (6.2%), Unigold (6.4%), Insti (11.4%) and SD Bioline (12.5%) had the least WP scores. All kits had a WP score in all participating centers (Table 1). Eastern (58.8%) followed by Central Uganda (53.8%) had significantly higher proportion of samples testing WP on any kit compared to Western (43.9%) and Northern Uganda (47.7%), table not shown. For all the kits evaluated, the exclusion of samples with WP bands improved the specificity and PPVs.

**Table 1:** Rapid test results by testing center

Rapid test kit	AIC				Mbarara				Kyamulibwa				Rakai				Lacor				Jinja				Soroti				All Centers			
	P	WP	N	Tot	P	WP	N	Tot	P	WP	N	Tot	P	WP	N	Tot	P	WP	N	Tot	P	WP	N	Tot	P	WP	N	Tot	P	WP	N	Tot
Determine *(r%)*	186	5	209	400	193	4	203	400	190	46	164	400	195	13	192	400	195	17	188	400	163	15	222	400	195	73	132	400	1317	173	1310	2800
	*46.5*	*1.3*	*52.3*		*48.3*	*1.0*	*50.8*		*47.5*	*11.5*	*41.0*		*48.8*	*3.3*	*48.0*		*48.8*	*4.3*	*47.0*		*40.8*	*3.8*	*55.5*		*48.8*	*18.3*	*33*		*47.0*	*6.2*	*46.8*	
HIVSav *(r%)*	152	38	210		180	18	202		20	183	197		59	144	197		121	87	192		91	84	225		30	166	204		653	720	1427	
	*38*	*9.5*	*52.5*		*45*	*4.5*	*50.5*		*5.0*	*45.8*	*49.3*		*14.8*	*36.0*	*49.3*		*30.3*	*21.8*	*48.0*		*22.8*	*21.0*	*56.3*		*7.5*	*41.5*	*51.0*		*23.3*	*25.7*	*51.0*	
Acon Triline *(r%)*	157	34	207	398	171	24	205		29	174	197		33	166	201		126	80	194		51	126	223		35	165	200		602	769	1427	2798
	*39.5*	*8.5*	*52.0*		*42.8*	*6.0*	*51.3*		*7.3*	*43.5*	*49.3*		*8.3*	*41.5*	*50.3*		*31.5*	*20.0*	*48.5*		*12.8*	*31.5*	*55.8*		*8.8*	*41.3*	*50.0*		*21.5*	*27.5*	*51*	
Unigold *(r%)*	168	21	211		185	8	207		166	37	197		194	4	202		183	16	201		144	31	225		135	61	204		1175	178	1447	
	*42*	*5.3*	*52.8*		*46.3*	*2*	*51.8*		*41.5*	*9.3*	*49.3*		*48.5*	*1*	*50.5*		*45.8*	*4*	*50.3*		*36*	*7.8*	*56.3*		*33.8*	*15.3*	*51*		*42.0*	*6.4*	*51.7*	
SD Bioline *(r%)*	141	47	212		161	40	199		126	73	201		179	17	204		169	37	194		124	52	224		111	85	204		1011	351	1438	
	*35.3*	*11.8*	*53*		*40.3*	*10*	*49.8*		*31.5*	*18.3*	*50.3*		*44.8*	*4.3*	*51*		*42.3*	*9.3*	*48.5*		*31*	*13*	*56*		*27.8*	*21.3*	*51*		*36.1*	*12.5*	*51.4*	
First Response *(r%)*	79	118	203		138	75	187		3	210	187		25	179	196		128	93	179		15	166	218		9	180	192		397	1021	1362	
	*19.8*	*29.5*	*50.8*		*34.5*	*18.8*	*46.8*		*0.8*	*52.5*	*46.8*		6.3	*44.8*	*49*		*32*	*23.3*	*44.8*		*3.8*	*41.6*	*54.6*		*2.4*	*47.2*	*50.4*		*14.3*	*36.7*	*49.0*	
Carestart *(r%)*	83	112	204	399	156	61	183		7	215	178		28	182	190		140	86	174		23	160	217		9	192	199		446	1008	1345	2799
	*20.8*	*28.1*	*51.1*		*39*	*15.3*	*45.8*		*1.8*	*53.3*	*45.8*		*7*	*45.5*	*47.5*		*35*	*21.5*	*43.5*		*5.8*	*40*	*54.3*		*2.3*	*48*	*49.8*		*15.9*	*36*	*48.1*	
Doublechek *(r%)*	110	82	208		139	72	189		43	162	195		182	20	198		156	48	195	399	36	37	126	199	7	101	167	275	673	522	1278	2473
	*27.5*	*20.5*	*52*		*34.8*	*18*	*47.3*		*10.8*	*40.5*	*48.8*		*45.5*	*5*	*49.5*		*39.1*	*12*	*48.9*		*18.1*	*18.6*	*63.3*		*2.6*	*36.7*	*60.7*		*27.2*	*21.1*	*51.7*	
Statpak *(r%)*	178	6	212	396	188	7	205		166	34	200		190	11	199		189	7	203	399	169	8	223		186	10	204		1266	83	1446	2795
	*45*	*1.5*	*53.5*		*47*	*1.8*	*51.3*		*41.5*	*8.5*	*50*		*47.5*	*2.8*	*49.8*		*47.4*	*1.8*	*50.9*		*42.3*	*2.0*	*55.8*		*46.5*	*2.5*	*51*		*45.3*	*3.0*	*51.7*	
Medinostics *(r%)*	113	77	210		179	16	205		57	141	202		147	51	202		172	24	203	399	82	91	227	*20.5*	33	161	206		783	561	1455	2799
	*28.3*	*19.3*	*51.3*		*44.8*	*4.0*	*51.3*		*14.3*	*35.3*	*50.5*		*36.8*	*12.8*	*50.5*		*43.1*	*6*	*50.9*		*22.8*	*56.8*			*8.3*	*40.3*	*51.5*		*28*	*20*	*52*	
Insti *(r%)*	175	13	210	398	187	6	207		90	110	200		190	10	200		193	9	198		121	55	224		78	116	206		1034	319	1445	2798
	*44*	*3.3*	*52.8*		*46.8*	*1.5*	*51.8*		*22.5*	*27.5*	*50*		*47.5*	*2.5*	*50*		*48.3*	*2.3*	*49.5*		*30.3*	*13.8*	*56*		*19.5*	*29*	*51.5*		*37*	*11.4*	*51.6*	

Total samples per site 400 except where indicated, r% stands for row percentage

Table 2 shows the performance of the blood based kits with WP taken as positive and after removal of the WP (No WP). Determine had the highest sensitivity of 99.3% and 99.2% with and without WP respectively. Insti had the second best sensitivity of 99.2% and 98.9% with and without WP respectively. Medinostics had the best specificity of 99.1 and 99.8 with and without WP respectively. Unigold had the best specificity when WP were taken as positive and 9 RTs had specificity of ≥90 after removal of WPs.

**Table 2:** Summary of the kit performance for the blood based kits

Rapid Test Kit	Sensitivity		Specificity		PPV		NPV		FPR		FNR	
	With WP as POS	After removal of WP	With WP as POS	After removal of WP	With WP as POS	After removal of WP	With WP as POS	After removal of WP	With WP as POS	After removal of WP	With WP as POS	After removal of WP
Determine	99.3	99.2	89.6	99.0	90.1	99.0	99.2	99.2	10.4	1.0	0.7	0.8
HIVSav	99.1	98.2	97.6	99.6	97.5	99.2	99.1	99.1	2.4	0.4	0.9	1.8
Acon	99.0	97.7	97.5	99.7	97.4	99.3	99.0	99.0	2.5	0.3	1.0	2.3
Unigold	98.9	98.7	98.6	99.6	98.6	99.5	98.9	98.9	1.4	0.4	1.1	1.3
SD Bioline	99.0	98.6	98.3	99.7	98.2	99.6	99.0	99.0	1.7	0.3	1.0	1.4
First Response	99.0	96.7	93.4	98.9	93.4	96.4	98.6	98.6	6.6	1.1	1.0	3.3
Carestart	99.0	97.0	92.1	98.4	92.3	95.2	99.0	99.0	7.9	1.6	1.0	3.0
Doublecheck	98.7	97.8	96.2	99.4	95.9	98.8	98.8	98.8	3.8	0.6	1.3	2.2
Statpak	98.8	98.7	98.4	99.5	98.3	99.4	98.9	98.9	1.6	0.5	1.2	1.3
Medinostics	98.9	98.1	99.1	99.8	99.1	99.6	98.9	98.9	0.9	0.2	1.1	1.9
Insti	99.2	98.9	98.2	99.8	98.9	99.7	99.2	99.2	1.1	0.2	0.8	1.1

Figures represent percentages, with weak positives taken as positives and after their removal

Table 3 shows the performance of the oral kits. Aware had the highest sensitivity of 97% when WP were included and 96.4 after removal of WP. On the other hand, Oraquick had the highest sensitivity of 96.5% when WPs were excluded which increased to 96.8% when WP were included. Oraquick and Aware had the highest specificity of 98.5% when WPs were included and the highest specificity of 99.2% when WPs were excluded.

**Table 3:** Summary of performance of the Oral kits

Rapid test kit	Sensitivity		Specificity		PPV		NPV		FPR		FNR	
	With WP as POS	After removal of WP	With WP as POS	After removal of WP	With WP as POS	After removal of WP	With WP as POS	After removal of WP	With WP as POS	After removal of WP	With WP as POS	After removal of WP
Oraquick	96.8	96.5	98.5	99.2	98.5	99.1	96.9	96.9	1.5	0.8	3.2	3.5
Aware	97	96.4	98.5	99.2	98.4	99	97.1	97.1	1.5	0.8	3	3.6
DPP	96.4	95.6	98.2	99	98.2	98.7	96.5	96.5	1.8	1	3.6	4.4

Figures represent percentages, with weak positives taken as positives and after their removal

After sensitivity and specificity analyses, it was observed that the best test kits that could be used as screening tests were Determine and Insti (with best sensitivity before and after removing results with WP bands). However, it was also observed that Medinostics had the “best” PPV of 99.1% before and 99.6% after the removal of results with a WP band. Medinostics also had the highest specificity of 99.1% before and 99.8% after removal of results with a WP band.

It was therefore decided to compare algorithms where Determine and Insti were the screening tests. Combinations of all kits that met the above criteria were then made and the performance (sensitivity, specificity, PPV, NPV, FPR, FNR) and cost of the different algorithms calculated. None of the 3 oral mucosal transudate kits were suitable for inclusion in an algorithm evaluation because of their low sensitivities.

Even though HIVsav passed the criteria for a screening kit, all algorithms in which it was included were eliminated because their sensitivities were lower than the current algorithm.

Based on the results in Tables 4, 18 algorithms had a performance better than the current national algorithm. The three algorithms indicated in Tables 4 and 5, 1) Determine + SD Bioline + Statpak; 2) Determine + Statpak + SD Bioline and 3) Determine + Statpak + Insti which performed better than the current algorithm and met the cost requirements are recommended as the new national algorithms. Algorithms containing Medinostics were all removed because the kit could not be procured by the study team.

**Table 4:** Summary of possible algorithms with a performance as good as or better than the current^a^

Rank	Screening	Confirmatory	Tie Breaker	Sensitivity	Specificity	PPV	NPV	FPR	FNR	Cost per test ($)
1	Insti	SDBioline	StatPak	99.2	99.4	99.4	99.2	0.6	0.8	4.61
2	Insti	Statpak	SD Bioline	99.2	99.4	99.4	99.2	0.6	0.8	4.61
3^b^	**Determine**	**SD Bioline**	**StatPak**	**99.2**	**99.1**	**99.0**	**99.2**	**0.9**	**0.8**	**1.43**
4^b^	**Determine**	**Statpak**	**SD Bioline**	**99.2**	**99.1**	**99.0**	**99.2**	**0.9**	**0.8**	**1.43**
5^b^	**Determine**	**Statpak**	**Insti**	**99.2**	**99.1**	**99.0**	**99.2**	**0.9**	**0.8**	**1.79**
6	Determine	Insti	StatPak	99.2	99.1	99.0	99.2	0.9	0.8	2.05
7	Insti	SD Bioline	Medinostics	99.1	99.4	99.4	99.1	0.6	0.9	4.61
8	Insti	Medinostics	SD Bioline	99.1	99.4	99.4	99.1	0.6	0.9	4.61
9	Insti	Medinostics	Statpak	99.1	99.4	99.4	99.1	0.6	0.9	4.61
10	Insti	StatPak	Medinostics	99.1	99.4	99.4	99.1	0.6	0.9	4.61
11	Insti	Statpak	Unigold	99.1	99.4	99.3	99.1	0.6	0.9	4.62
12	Insti	Unigold	Statpak	99.1	99.4	99.3	99.1	0.6	0.9	4.66
13	Determine	Medinostics	Statpak	99.1	99.2	99.2	99.1	0.8	0.9	1.43
14	Determine	StatPak	Medinostics	99.1	99.2	99.2	99.1	0.8	0.9	1.43
15	Determine	Medinostics	Insti	99.1	99.1	99.1	99.1	0.9	0.9	1.78
16	Determine	Insti	Medinostics	99.1	99.1	99.1	99.1	0.9	0.9	2.05
17	Determine	SD Bioline	Insti	99.1	99.0	99.0	99.1	1.0	0.9	1.78
18	Determine	Insti	SD Bioline	99.1	99.0	99.0	99.1	1.0	0.9	2.05
19^c^	*Determine*	*Statpak*	*Unigold*	*99.1*	*98.9*	*98.9*	*99.1*	*1.1*	*0.9*	*1.50*
20	Determine	Unigold	Statpak	99.1	98.9	98.9	99.1	1.1	0.9	1.54
21	Determine	Unigold	Insti	99.1	98.9	98.9	99.1	1.1	0.9	1.89
22	Determine	Insti	Unigold	99.1	98.9	98.9	99.1	1.1	0.9	2.11

^a^The order of ranking followed the priority of: 1-sensitivity, 2-specificity, 3-PPV, 4-NPV, 5-FPR, 6-FNR, 7-cost; ^b^and bolded are the proposed algorithms;

^c^In italics is current algorithm

**Table 5:** Summary of possible algorithms with a performance as good as or better than the current algorithm ranked in order of cost^a^

Rank	Screening	Confirmatory	Tie Breaker	Sensitivity	Specificity	PPV	NPV	FPR	FNR	Cost per test ($)
1^b^	**Determine**	**SD Bioline**	**StatPak**	**99.2**	**99.1**	**99**	**99.2**	**0.9**	**0.8**	**1.43**
2	Determine	Medinostics	Statpak	99.1	99.2	99.2	99.1	0.8	0.9	1.43
3^b^	**Determine**	**Statpak**	**SD Bioline**	**99.2**	**99.1**	**99**	**99.2**	**0.9**	**0.8**	**1.43**
4	Determine	StatPak	Medinostics	99.1	99.2	99.2	99.1	0.8	0.9	1.43
5^c^	*Determine*	*Statpak*	*Unigold*	*99.1*	*98.9*	*98.9*	*99.1*	*1.1*	*0.9*	*1.50*
6	Determine	Unigold	Statpak	99.1	98.9	98.9	99.1	1.1	0.9	1.54
7	Determine	SD Bioline	Insti	99.1	99.0	99.0	99.1	1.0	0.9	1.78
8	Determine	Medinostics	Insti	99.1	99.1	99.1	99.1	0.9	0.9	1.78
9^b^	**Determine**	**Statpak**	**Insti**	**99.2**	**99.1**	**99.0**	**99.2**	**0.9**	**0.8**	**1.79**
10	Determine	Unigold	Insti	99.1	98.9	98.9	99.1	1.1	0.9	1.89
11	Determine	Insti	Medinostics	99.1	99.1	99.1	99.1	0.9	0.9	2.05
12	Determine	Insti	SD Bioline	99.1	99.0	99.0	99.1	1.0	0.9	2.05
13	Determine	Insti	StatPak	99.2	99.1	99.0	99.2	0.9	0.8	2.05
14	Determine	Insti	Unigold	99.1	98.9	98.9	99.1	1.1	0.9	2.11
15	Insti	SD Bioline	Medinostics	99.1	99.4	99.4	99.1	0.6	0.9	4.61
16	Insti	Medinostics	SD Bioline	99.1	99.4	99.4	99.1	0.6	0.9	4.61
17	Insti	SD Bioline	StatPak	99.2	99.4	99.4	99.2	0.6	0.8	4.61
18	Insti	Medinostics	Statpak	99.1	99.4	99.4	99.1	0.6	0.9	4.61
19	Insti	Statpak	SD Bioline	99.2	99.4	99.4	99.2	0.6	0.8	4.61
20	Insti	StatPak	Medinostics	99.1	99.4	99.4	99.1	0.6	0.9	4.61
21	Insti	Statpak	Unigold	99.1	99.4	99.3	99.1	0.6	0.9	4.62
22	Insti	Unigold	Statpak	99.1	99.4	99.3	99.1	0.6	0.9	4.66

^a^The order of ranking followed the priority of: 1- Cost, 2-sensitivity, 3-specificity, 4-PPV, 5-NPV, 6-FPR, 7-FNR; ^b^ In bold are the proposed algorithms;

^c^In italics current algorithm

### Receiver operating curves

The ROC observed showed no statistically significant differences between the three selected algorithms and the currently used one. Determine + Statpak + SDBio to Determine + Statpak + Unigold, (*p* = 0.31); Determine + SDBio + Statpak to Determine + Statpak + Unigold, (*p* = 0.31); Determine + Statpak + Insti to Determine + Statpak + Unigold (*p* = 0.25). The ROC area for the three new algorithms was marginally wider when compared to that generated for the current national algorithm.

### Inter-reader variability

Analyses done at three sites, Rakai, Jinja and Soroti showed that the inter-reader variability between two technicians for all the tests was low, in most cases below 1%, meaning that there were similarities in the interpretation of results (Table 6), exceptions were Jinja where for Carestart this was 1.26 and 2.01 for Double check and at Soroti where for Determine this was 2.42% and for Insti 3.63%. The majority of differences happened when one reader interpreted a result as having a weak positive band while another interpreted it as being truly positive. There were also cases where one reader interpreted result as having WP band while the other recorded as negative result.

**Table 6:** Inter reader variability

Rapid test kit	Rakai variability (n, %)	Jinja variability (n, %)	Soroti variability (n, %)	Total variability (n, %)
Determine	1, 0.25	1, 0.25	6, 2.42	8, 0.76
HIVSav	3, 0.75	0, 0	0, 0	3, 0.29
Acon Triline	2, 0.50	0, 0	2, 0.81	4, 0.38
Unigold	1, 0.25	1, 0.25	2, 0.81	4, 0.38
SD Bioline	1, 0.25	1, 0.25	2, 0.81	4, 0.38
First Response	3, 0.75	3, 0.76	1, 0.43	7, 0.68
Carestart	0, 0	5, 1.26	0, 0	5, 0.59
Doublechek	0, 0	4, 2.01	0, 0	4, 0.57
Statpak	1, 0.25	3, 0.75	0, 0	4, 0.38
Medinostics	0, 0	1, 0.25	0, 0	1, 0.10
Insti	1, 0.25	2, 0.50	9, 3.63	12, 1.15

### Antigens and technology used

Since it is advisable to use in combinations tests that use different antigens and technology, this was also examined for the kits used in the proposed three algorithms as shown in Table 7. One manufacturer did not provide this information.

**Table 7:** Antigens and technology used in the selected kits

Kit	Antigen	Technology
Determine	Recombinant (Rec) HIV-1 (gp120 and gp41), Rec HIV-2 (gp36)	Lateral flow immunochromatography
SD Bioline	Rec HIV-1 (gp 41 and p24) and RecHIV-2 (gp 36)	Lateral flow immunochromatography
Statpak	No information	Lateral flow immunochromatography
Insti	Rec HIV-1 (gp 41) and Rec HIV-2 (gp 36)	Flow through immunochromatography

### Phase II

In phase II, sites tested a total of 2398 samples. A total of 901 site-specific results were included in the comparison and were compared to the NRL plasma EIA results. Results from AIC, TASO Entebbe and Lacor Hospital were 100% concordant with the NRL results. Concordance between results at the TASO Masaka site and NRL was 96.7% with 5 sample results being discordant with EIA results, 2 for one algorithm (Determine, Statpak, SD Bioline) and 3 for the other (Determine, Statpak and Insti). Two of the samples resulted in false negative EIA results while 3 resulted in false positive EIA results. The discordant results were all from one operator who joined later in the study and were attributed to inadequate training.

## Discussion

This evaluation was necessary because there were indications that the current national algorithm which has been in use for quite some time may need to be changed. In addition, several new HIV RTs kits had been developed that may be better in terms of cost or ease of use if used as part of the national algorithm. There was also a demand to provide alternative algorithms for better service delivery. To achieve this, we used a more rigorous CDC/WHO recommended evaluation method compared to that which was used earlier.

In our study, the performance of the individual RTs varied. While the sensitivity of all kits except for four was within that recommended by WHO i.e. (≥ 99%) [17], the specificity of six kits was lower than the recommended ≥98%, if the WP bands are scored as positive leading to low PPV. A similar observation was reported before with some of these kits i.e. Determine and Unigold [14, 18]. There is a recent study that has also shown poor performance of some RTs in sub-Saharan Africa than the WHO evaluations [19], and this was not attributed to weak bands, which were rarely reported in their study. There are varied reasons for the performance differences, for instance, it was reported that the sensitivity of Insti could be enhanced by its ability to detect HIV gp41 IgM antibodies [20].

The performance of the oral based kits was lower than the blood based ones, especially their sensitivity and NPV which fell below the recommended WHO performance targets. We are aware that a few other studies in Africa have shown good performance of these oral based kits [21–23] and their use in self-testing is being promoted. Such a policy is also being developed in Uganda and our recommendation is to emphasize the need to perform other confirmatory tests based on blood assays after oral self-testing. Since all those involved in testing were well trained we do not think this poor performance of the oral based kits was due to the testing being performed by nurse counsellors as opposed to laboratory technician who performed the blood based RTs.

We have identified three algorithms whose cost is either lower or within 20% the price of the current one and with better performance. We recommend these algorithms to be adopted by the Ministry of Health (MOH) as the national algorithms for the country. The above approach gives providers a choice of algorithms that can be used especially if there are procurement challenges such as those we observed when one of the kits was temporarily withdrawn. We however recognize the challenges of moving from using one algorithm to three. There will be a need to have training and understanding new concepts including the possibility of interchanging the confirmatory and the tie breaker kits since two of the algorithms can use these interchangeably i.e. Determine + SD Bioline + Statpak or Determine + Statpak + SD Bioline. If the MOH eventually decides to reduce to two algorithms these two would be preferred since it simplifies training and procurement. On the other hand, if it is recommended to stick to only one algorithm as currently, and from the initial discussions this appears to be the preferred option, then the algorithm to recommend would be Determine + Statpak + SD Bioline, the reason being this would be very easy to introduce since there is only one change from the current algorithm, replacing Unigold as the tie breaker with SD Bioline. Our study therefore provides the policy makers with a number of options.

Though the ROC for the three selected algorithms did not significantly differ from the currently used one, the above performance in terms of Se, Sp, NVP and PPV met the pre-set performance requirements to guide the selection.

We also recommend that all algorithms that performed better than the current one are made available as alternatives for the private sector where choice is largely based on performance rather than cost. Currently different test algorithms are being used in the private sector guided by availability and unfortunately, some of these algorithms have not been evaluated locally. Our recommendation will provide the private sector with a number of alternatives; some of these have very high performance only limited by cost which may not be an issue for some clients. The challenge is that these are too many algorithms and training and QC programmes could be difficult to implement in such situations yet they are important components of HCT and RT scaling up [24].

From our study, we propose that kits being recommended should be used following the manufacturers criteria, scoring both strong and WP bands as positive. When serial testing as being proposed is followed, the performance of these kits would be improved and the intensity of the bands would be less of an issue. We however recognize the fact that reading/interpretation of RT strips is subjective and depends on a number of factors not least, the available light and the reader’s visual acuity. There are now efforts by manufacturers to provide automated readers that would address this subjectivity.

Our study did not identify significant geographical regional differences in the frequency of WP bands in Uganda possibly ruling out a local environment factor as responsible for these WP bands.

The screening tests recommended are very sensitive but with low specificity. Due to this, service providers should avoid giving final positive test results based on the screening test results alone as often reported, which could be false positive.

The use of highly specific confirmatory test ensures that a confirmed positive sample is likely to be a true positive. With a highly specific tie-breaker any concordance positive result with the confirmatory test would provide a definitive result. On the other hand, a rare discordance between a tie breaker and an equally specific confirmatory test would warrant a client to have a repeat test after 14 days, a strategy recommended by WHO [17] in high prevalence settings which we propose to adopt under the new national Algorithm. If the rate of HIV-inconclusive results is high, additional efforts to assure quality should be made, and the selection of assays might be reconsidered.

HIV misdiagnosis has recently been reported [25], raising concerns about RT algorithms. Our study however on the contrary has shown that with carefully selected RTs, we can achieve the WHO recommended testing algorithms performance. By further adopting the above additional WHO recommendations for high HIV prevalence settings, we expect increased test results accuracy. None of our best performing algorithms was tested in the Kosack study.

The rapid tests recommended had acceptable inter-reader variability within the WHO recommended < 5% [2], the minor differences were largely as a result of recording the intensity of the bands as either WP or being truly positive an issue which can be minimized if we follow the manufacturer’s guidelines and record all bands as positive. The finger stick testing at the POC was performed at acceptable standards except for one center in Masaka and this was by one individual, as reported above, this was attributed to poor training of this person who joined later in the study. We therefore recommend an active training and QC programme if we are to have an efficient HCT programme.

Since the oral based kits individually did not perform as well as the blood based ones, they are not recommended to be part of the national algorithm.

This HIV RT evaluation, has allowed our NRL to collect and store specimens from different parts of Uganda that will in future be available for QA/QC of new tests that come on market.

We recognize that the kits evaluated are all 3rd generation assays and currently new 4th generation tests have come on the market [6]. The advantage of these new kits is the early detection of recent seroconverters due to the inclusion of the p24 antigen detection. The performance of these new tests for the detection of acute infection are however still questionable [5, 6]. In this evaluation, we also explored the performance of one such kit i.e. the Alere Determine HIV-1/2 Ag/Ab Combo (data not shown). It had a good sensitivity equivalent to the 3rd generation kit, and a considerably better specificity. We were however unable to evaluate its accuracy in the detection of early seroconverters and therefore we could not make meaningful recommendations on its use.

Our study has a number of strengths; it is the largest rapid test evaluation in Uganda in terms of subject numbers and test kits. It is the first that has followed both the CDC/WHO recommended phase I and II approaches which in addition to laboratory, include POC field evaluations by non-laboratory workers. The study evaluated both the blood based and three commercially available oral based kits. Furthermore, the study followed very high QA/QC checks supervised by a highly experienced team with some of the laboratories used having various accreditations such as ISO 15189 and College of American Pathologist (CAP). The other strength of our study was that it was carried out with continuous consultations with the MOH and the C17 committee which advises on issues of HTC. Their recommendations were therefore taken into consideration.

The study had some challenges. There were difficulties in the procurement of some kits. As mentioned earlier, one very good kit Medinostics HIV1/2 Gold Rapid Screen Test was dropped because it could not be procured. The SD Bioline rapid HIV test was temporarily withdrawn by The U.S. President’s Emergency Plan for AIDS Relief (PEPFAR) and removed from the USAID list of approved rapid HIV test kits. During the period of the evaluation and as mentioned earlier Determine changed manufacture from Abbott to Alere Determine, which came on market. We had to evaluate this new test before moving to phase II. The study had planned to perform inter-lab evaluation but this was not possible due to logistical challenges. Finally, there were also some pressures from some manufacturers and suppliers to include their tests in the evaluation.

Like a few other studies in Africa that followed the CDC/WHO recommendations, it was expensive and took long to complete due to different factors including those mentioned above.

The limitations of this study included the fact that the criteria for a fresh blood sample may not have met the manufacture’s recommendations for some kits investigated in phase I of the study due to some delays in transfer of samples from the field to the laboratory. The evaluation of test kits using venous blood sample in phase I may not mimic that using finger stick in the field due to the time difference between collection and testing and also due to the possible interference of the anticoagulant. Whereas it was preferable to use samples from volunteers whose HIV status was unknown and avoid selection bias, the total sample size required to collect all the HIV positives using this approach was going to make the study very costly and hence samples were collected from both known and unknown HIV positive and negative study participants. We decided to use the CDC/WHO guidelines for the sample size as using area specific HIV prevalence resulted in a big sample that could not be used because of budget limitations. It was not logistically possible to evaluate all the 18 algorithms that performed better than the current algorithm in phase II, hence the performance that we report for fifteen of these is limited to the laboratory evaluation (phase I). Finally, the inter observer agreement using finger stick whole blood was not possible between the field site.

## Conclusion

In this first evaluation of a national HIV RT algorithm in Uganda using the CDC/WHO recommended method, we have come up with three algorithms we recommend for public or Government procurement based on accuracy and cost, i.e. Determine + SD Bioline + Statpak; Determine + Statpak + SD Bioline and Determine + Statpak + Insti. If it is recommended to stick to only one algorithm as currently, then the algorithm to recommend would be Determine + Statpak + SD Bioline, replacing Unigold as the tie breaker with SD Bioline. We further recommend that all the 18 algorithms that have shown better performance than the current one are all made available to the private sector especially where cost is not a limiting factor.

## Appendix

### Calculation of Algorithm Costs

The procedure below was used to estimate the cost of each algorithm.

*Algorithm Cost = Screening kit cost + Confirmatory kit Cost + Tie breaker kit Cost.*

*Following National Guidelines, if a sample turns out to be Negative on the screening test, then the cost of testing that sample would only be equal to the screening test kit cost for a sample. If the sample turns out to be Positive, then you would use a confirmatory test. If the confirmatory test turns out to be positive then the cost of test for that sample would be equal to cost per sample for screening + confirmatory test. If, however the screening test result is positive, and the confirmatory test result is negative then you would need a tie breaker. The cost of testing such a sample would then be the.*

*Screening kit cost + Confirmatory kit cost + Tie Breaker kit cost.*

*Example using three samples A, B, and C.*

*The Algorithm under review consists of Kits X (screening kit), Y (confirmatory kit) and Z (Tie Breaker).*

*X Costs 10 Dollars a test.*

*Y costs 15 dollars a test.*

*Z Costs 20 Dollars a test.*

*Sample A.*

*On testing Sample A using the screening kit X it turns out NEGATIVE. Following National Guidelines, the testing would be stopped and results reported. So the Total Cost of testing Sample A would be 10 $.*

*Sample B.*

*On testing sample B using screening kit X it turns out as POSITIVE. This requires a Confirmatory test (Y). On testing it using Kit Y it turns out POSITIVE. The testing would stop and results would be reported. The total cost of testing sample B would be 10 + 15 = 25 $.*

*Sample C.*

*On testing sample C using screening kit X it turns out as POSITIVE. This requires a Confirmatory test (Y). On testing it using Kit Y it turns out as NEGATIVE. This would then require a tie breaker (Z). On testing, it turns out as NEGATIVE or POSITIVE. Then results that are reported would be NEGATIVE or POSITIVE in conformity to what the tie breaker kit result was. The cost of testing sample C would be 10 + 15 + 20 = 45$.*

In determining the cost of an algorithm, we need to put into consideration the HIV prevalence in the country. While all samples would be tested on the screening test, the number of samples that eventually go for confirmatory and tie breaker testing will depend on the HIV prevalence (About 7.3% if we take this as the national average prevalence) and to a lesser extent the sensitivity and specificity of the kits used.

*Screening cost = Cost of testing all samples using the screening kit.*

*Confirmatory cost = Cost of testing true positives using the confirmatory kit + Cost of testing screening kit false positives using the confirmatory kit-Cost of testing screening kit false negatives using the confirmatory kit.*

*Tie breaker cost = Cost of testing confirmatory kit false negatives using the tie breaker kit + cost of testing screening kit false positives using the tie breaker kit-Cost of testing screening and confirmatory kit false positives.*

Kit costs were obtained from the USAID Source and Origin Waiver: Procurement Information Document, fifth Edition, 2009 and the WHO 2013 prices. This document put the cost of Determine at $1.2, Medinostics $0.85, Statpak $0.9, Unigold $1.5, Insti $4.53, and SD Bioline $0.85.

Example 1

Suppose you want to test 10,000 samples using the current algorithm in a population with an HIV prevalence of 7.3%.

The total no. of HIV positives =0.073 × 10,000 = 730.

The total no. of HIV negatives = 10,000–730 = 9270.

Using the formular above and figures in Table 1.

Screening cost = 10,000 × 1.2 = $12,000.

Confirmatory cost = (730 × 0.9) + (0.104 × 9270) × 0.9-(0.007 × 730) × 0.9 = 1520.

Tie breaker cost = (0.012 × 730) × 1.5 + (0.104 × 9270) × 1.5-(0.104x9270x0.016) × 1.5 = 1436.

Total cost = 12,000 + 1520 + 1436 = 14,956.

Algorithm cost per sample = 14,956/10,000 = $1.50.

Example 2

If prevalence is 30%,

Total no of HIV positives will = 0.3 × 10,000 = 3000.

Total no of negatives = 10,000–3000 = 7000.

Screening cost = 10,000 × 1.2 = $12,000.

Confirmatory cost = (3000 × 0.9) + (0.104 × 7000) × 0.9-(0.007 × 3000) × 0.9 = 3336.

Tie breaker cost = (0.012 × 3000) × 1.5 + (0.104 × 7000) × 1.5-(0.104x7000x0.016) × 1.5 = 1129.

Total cost = 12,000 + 3336 + 1129 = 16,465.

Algorithm cost per sample = 16,501/10,000 = $1.65.

## Abbreviations

AIC: AIDS Information Center
CAP: College of American Pathologists
CDC: Centers for Disease Control and Prevention
EDTA: ethylenediaminetetraacetic acid
EIAs: Enzyme immunoassays
FDA: Food and Drug Administration
FNR: False negative rate
FPR: False positive rate
HIVST: HIV self-testing
HTC: HIV testing and counselling
mls: milliters
MOH: Ministry of Health
MRC: Medical Research Council
NPV: Negative Predicative Value
NRL: National Reference Laboratory
PEPFAR: US President’s Emergency Plan for AIDS Relief
POC: Point of Care
PPV: Positive Predictive Value
QA: Quality assurance
QC: Quality Control
RHSP: Rakai Health Sciences Program
ROC: Receiver Operating Curves
RT: Rapid test
Se: Sensitivity
SOP: Standard Operating Procedure
Sp: Specificity
SST: Serum separator tubes
TASO: The AIDS Support Organization
UAIS: Uganda AIDS Indicator Survey
UNAIDS: The Joint United Nations Programme on HIV/AIDS
USAID: United States Agency for International Development
UVRI: Uganda Virus Research Institute
WHO: World Health Organization
WP: Weak positives
